# Influence of Benz[*a*]anthracene on Bone Metabolism and on Liver Metabolism in Nibbler Fish, *Girella punctata*

**DOI:** 10.3390/ijerph17041391

**Published:** 2020-02-21

**Authors:** Mohamed I. Zanaty, Niina Sawada, Yoichiro Kitani, Hossam F. Nassar, Hamada M. Mahmoud, Kazuichi Hayakawa, Toshio Sekiguchi, Shouzo Ogiso, Yoshiaki Tabuchi, Makoto Urata, Hajime Matsubara, Yutaka Takeuchi, Atsuhiko Hattori, Ajai K. Srivastav, Thumronk Amornsakun, Nobuo Suzuki

**Affiliations:** 1 Faculty of Postgraduate Studies for Advanced Sciences, Beni-suef University, Beni-Suef 62511, Egypt; zanaty012@yahoo.com (M.I.Z.); hossamnassarnrc@gmail.com (H.F.N.); 2 Noto Marine Laboratory, Institute of Nature and Environmental Technology, Division of Marine Environmental Studies, Kanazawa University, Noto-cho, Ishikawa 927-0553, Japan; yki@se.kanazawa-u.ac.jp (Y.K.); t-sekiguchi@se.kanazawa-u.ac.jp (T.S.); shozoogiso@se.kanazawa-u.ac.jp (S.O.); glandiceps@yahoo.co.jp (M.U.); 3 School of Natural System, College of Science and Engineering, Kanazawa University, Kakuma-machi, Kanazawa city, Ishikawa 920-1192, Japan; nobuosuzuki1964@gmail.com; 4 Faculty of Science, Beni-Suef University, Beni-Suef 62511, Egypt; mhmada@aucegypt.edu; 5 Low Level Radioactivity Laboratory, Institute of Nature and Environmental Technology, Kanazawa University, Nomi city, Ishikawa 923-1224, Japan; hayakawa@p.kanazawa-u.ac.jp; 6 Division of Molecular Genetics Research, Life Science Research Center, University of Toyama, Sugitani, Toyama 930-0194, Japan; ytabu@cts.u-toyama.ac.jp; 7 Institute of Noto SATOUMI Education Research, Noto-cho, Ishikawa 927-0553, Japan; 8 Noto Center for Fisheries Science and Technology, Kanazawa University, Ossaka, Noto-cho, Ishikawa 927-0552, Japan; matsu@se.kanazawa-u.ac.jp (H.M.); yutaka@se.kanazawa-u.ac.jp (Y.T.); 9 Department of Biology, College of Liberal Arts and Sciences, Tokyo Medical and Dental University, Ichikawa city, Chiba 272-0827, Japan; ahattori.las@tmd.ac.jp; 10 Department of Zoology, D.D.U. Gorakhpur University, Gorakhpur 273-009, India; ajaiksrivastav@hotmail.com; 11 Fisheries Technology Program, Faculty of Science and Technology, Prince of Songkla University, Pattani 94000, Thailand; thumronk.a@psu.ac.th

**Keywords:** benz[*a*]anthracene, fish scales, osteoblasts, osteoclasts, calcium, inorganic phosphorus, liver metabolism, marine fish

## Abstract

It has been reported that spinal deformity was induced in developing fish by the addition of polycyclic aromatic hydrocarbons (PAHs). To examine the mechanism of the disruption of fish bone metabolism, the effect of benz[*a*]anthracene (BaA), a kind of PAH, on plasma calcium, inorganic phosphorus, osteoblasts, and osteoclasts was investigated in this study. We also measured several plasma components to analyze the toxicity of BaA on other metabolisms. BaA (1 or 10 ng/g body weight) was intraperitoneally injected (four times) into nibbler fish during breeding, for 10 days, and it was indicated, for the first time, that injecting high doses of BaA to nibbler fish induced both hypocalcemia and hypophosphatemia. Furthermore, in the scales of nibbler fish treated with high doses of BaA, both osteoclastic and osteoblastic marker messengerRNA (mRNA) expressions decreased. These results are a cause of disruption of bone metabolism and, perhaps, the induction of spinal deformities. In addition, we found that total protein, metabolic enzymes in the liver, total cholesterol, free cholesterol, and high-density lipoprotein cholesterol levels significantly decreased in BaA-injected fish. These results indicate that BaA may affect liver diseases and emphasize the importance of prevention of aquatic PAH pollution.

## 1. Introduction

Polycyclic aromatic hydrocarbons (PAHs) are a series of organic compounds that are atmospheric environmental pollutants derived from petroleum and produced by the incomplete combustion of fossil fuel, wood, and other organic materials [1], as well as cigarette smoke [2]. It has been reported that PAHs affect bone metabolism in mammals. For example, PAHs (benzo[*a*]pyrene (BaP); 7,12-dimethylbenz[*a*]anthracene (DMBA)) contained in cigarette smoke induced a loss of bone mass and bone strength, possibly through an increase in bone turnover [2]. In humans, furthermore, associations between urinary PAH content and bone mass density were stronger for postmenopausal women when compared with the premenopausal group [3]. Atmospheric PAHs seem to influence bone metabolism in terrestrial animals, including humans.

In the aquatic environment as well as the atmospheric environment, aquatic PAH contamination derived from storm water runoff and atmospheric deposition is evident [1,4] and bone deformity induced by PAHs has been observed in pacific herring, pink salmon and zebrafish [5,6], although the underlying mechanism has not yet been fully elucidated. Therefore, we should be giving greater attention to bone metabolism in aquatic animals such as fish.

On the other hand, teleosts have a calcified organ—scales—that possesses osteoclasts, osteoblasts, and a calcified bone matrix [7,8,9,10,11]. Fish scales are a potential internal calcium reservoir other than vertebral bone [7,12]. In the case of mercury, a significant co-relationship between mercury levels in the scales and in the muscles was reported in the largemouth bass [13], although mercury did not accumulate in the vertebral bone of the fish [14]. Furthermore, fish scales have been used for reconstructing past contamination in aquatic systems [15]. This indicates that scales are more active than vertebral bone in bone metabolism. In addition, we have previously found an estrogenic effect on the mono-hydroxylated form of benz[*a*]anthracene (BaA) [16], but not on that of BaP, and reported that mono-hydroxylated BaA suppressed osteoblastic and osteoclastic activities in goldfish scales [17].

Thus, in the present study, we examined plasma calcium (Ca) and inorganic phosphorus (Pi) levels and both scale osteoclastic and osteoblastic activities after injecting a low level of benz[*a*]anthracene (BaA) (1 or 10 ng/g body weight) into marine teleosts, nibbler fish (*Girella punctata*). Furthermore, we measured several plasma components to analyze the toxicity of BaA on other metabolisms.

The present study is the first to demonstrate that BaA decreases both plasma Ca and Pi levels in marine teleosts resulting from the influence of the osteoclasts and osteoblasts of nibbler fish scales. In addition, our data suggest that BaA induces liver diseases in addition to bone diseases.

## 2. Materials and Methods

### 2.1. Animals

The nibbler fish (*Girella punctata*) is a member of the Kyphosidae family and is a seawater fish distributed in the warm shallow waters of East Asia. It is easy to collect by fishing around our research institute and for this reason, we used it in the present study. Nibbler fish (both sexes, n = 24, 54.5 ± 3.5 g, around 1 to 2 years old) were captured by fishing in Tsukumo Bay of the Noto Peninsula (Ishikawa Prefecture). After acclimation for approximately 2 weeks, these fish were used in the present experiments.

All experimental procedures were conducted in accordance with the Kanazawa University Guide for the Care and Use of Laboratory Animals.

### 2.2. Effects of BaA on Plasma Ca and Pi Levels in Nibbler Fish

Nibbler fish were divided into three groups (eight individuals each) and anesthetized with 0.04% of a 2-phenoxyethanol (FUJIFILM Wako Pure Chemical Corporation, Osaka, Japan), and BaA (FUJIFILM Wako Pure Chemical Corporation) (low dose: 1 ng/g of body weight, n = 8; high dose: 10 ng/g body weight, n = 8) was injected intraperitoneally. These concentrations were determined based on the sea water which is highly contaminated with PAHs (total PAHs: 0.99–1.36 ng/mL) [18]. The injection was performed four times (on days 1, 3, 6, and 9). BaA was first dissolved in dimethyl sulfoxide (DMSO), and then distilled water was added to the DMSO solution so that the DMSO concentration became 0.5%. Nibbler fish in the control group (n = 8) were injected with 0.5% DMSO solution in the same manner as were the experimental nibbler fish. These fish were kept in one aquarium per respective treatment for 10 days at 26 °C under a 12 h light/12 h dark cycle while adding a small amount of natural seawater. The amount of PAHs in the seawater used in the present study was less than 2 pg/mL [19]. The values of pH and the salinity of the seawater were stable at around 8.25–8.35 and 33.0–33.5 practical salinity units, respectively. During the experimental periods, fish were fed every morning. Ten days after breeding, these fish were anesthetized again with 0.04% of a 2-phenoxyethanol. Blood samples from anesthetized nibbler fish were collected from their caudal vessels using a heparinized syringe. The collected blood was put into a 1.5 mL tube. Thereafter, the tube was centrifuged at 15,000 rpm for 3 min. The separated plasma was immediately frozen and kept at –80 °C until use. The plasma total Ca levels (mg/dL) were determined using an assay kit (Calcium E, FUJIFILM Wako Pure Chemical Corporation) as described in Suzuki et al. (2017) [20] and Sato et al. (2017) [21]. The plasma Pi levels (mg/dL) were measured using an assay kit (IP-II, Kyowa MEDEX Co., Ltd., Tokyo, Japan). Ten days after BaA injection, scales on the right side were extracted from anesthetized nibbler fish to examine the influences of BaA on the osteoblasts and osteoclasts. The collected scales were frozen at –80 °C for messengerRNA (mRNA) analysis.

### 2.3. Osteoblastic and Osteoclastic Marker mRNA Expression in BaA-Treated Nibbler Fish Scales

Total RNAs were prepared from nibbler fish scales (in all three groups, n = 8) kept in one aquarium per respective treatment using a total RNA isolation kit (NucleoSpin RNA II, Takara Bio Inc., Otsu, Japan). Complementary DNA synthesis was performed using a kit (PrimeScript™ II 1st strand cDNA Synthesis Kit, Takara Bio Inc., Kusatsu, Japan). Gene-specific primers for matrix metalloproteinase-9 (MMP-9) [21] and collagen, type 1, α1 (COL1A1) [22] are indicated in Table 1. Elongation factor-1α (EF-1α) cDNA was amplified using a primer set [22] (Table 1). The PCR amplification was analyzed using a real-time PCR apparatus (Mx3000p, Agilent Technologies, Santa Clara, CA, USA) [21,23]. The annealing temperature of MMP-9, COL1A1, and EF-1α was 60 °C. The MMP-9 and COL1A1 mRNA levels were normalized to the EF-1α mRNA level [24].

### 2.4. Measurement of Plasma Components in BaA-Injected and Untreated Nibbler Fish

In each group (n = 8), plasma samples in the BaA-treated or untreated nibbler fish were sent to a commercial vendor (Oriental Yeast Co., Ltd., Tokyo, Japan) for analysis. Total protein, albumin, alkaline phosphatase (ALP) activity, lactate dehydrogenase (LDH) activity, aspartate transaminase (AST) activity, leucine aminopeptidase (LAP) activity, total-cholesterol (T-CHO), free-cholesterol (F-CHO), ester type-cholesterol (E-CHO), and triglyceride (TG) were measured using several kits (FUJIFILM Wako Pure Chemical Corporation). Kits from Sekisui Medical Co. were used to determine low-density lipoprotein cholesterol (LDL-C) and high-density lipoprotein cholesterol (HDL-C). Sodium (Na), chlorine (Cl), and potassium (K) in the plasma of BaA-treated or untreated nibbler fish were measured by an ion electrode method with a Hitachi 7180 automatic analyzer (Hitachi High Technologies Corporation, Tokyo, Japan).

### 2.5. Statistical Analysis

All results are expressed as the means ± standard error (SE). The statistical significance between the control and experimental groups was assessed using a one-way ANOVA followed by Dunnett’s test or Student’s *t*-test. In all cases, the selected significance level was *p* < 0.05.

## 3. Results

### 3.1. Effects of BaA on Plasma Ca and Pi Levels in BaA-Treated or Untreated Nibbler Fish

**Figure 1:**
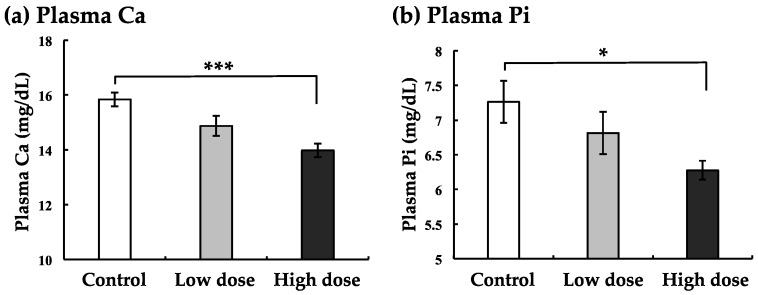
Effects of BaA on plasma calcium (Ca) (**a**) and inorganic phosphorus (Pi) (**b**) levels in BaA-treated or untreated nibbler fish. BaA (1 or 10 ng/g body weight) was injected intraperitoneally (four times) into nibbler fish for 10 days during breeding. Thereafter, plasma Ca and Pi levels were examined in the control and experimental groups. * and *** indicate statistically significant differences at *p* < 0.05 and *p* < 0.001, respectively, from the values in the control scales (n = 8). The detection limits of Plasma Ca and Pi were 0.4 and 0.04 mg/dL, respectively.

Figure 1a indicates the result of plasma Ca levels after intraperitoneal BaA injection. BaA (low dose: 1 ng/g of body weight) tended to decrease. The higher dose (10 ng/g of body weight) of BaA induced significant hypocalcemia.

Furthermore, plasma Pi levels decreased with the low dose of BaA treatment, although there was not a significant difference between the experimental and control groups (Figure 1b). In the case of the higher dose, plasma Pi levels decreased significantly (Figure 1b).

### 3.2. Effects of BaA on Osteoclastic and Osteoblastic Marker mRNA Expression in The Scales of BaA-Treated or Untreated Nibbler Fish

At the higher dose (10 ng/g of body weight), BaA induced significant hypocalcemia and hypophosphatemia. Therefore, we examined the effect of BaA at the high dose on osteoclasts and osteoblasts of the scales of BaA-injected nibbler fish in an in vivo experiment.

**Figure 2:**
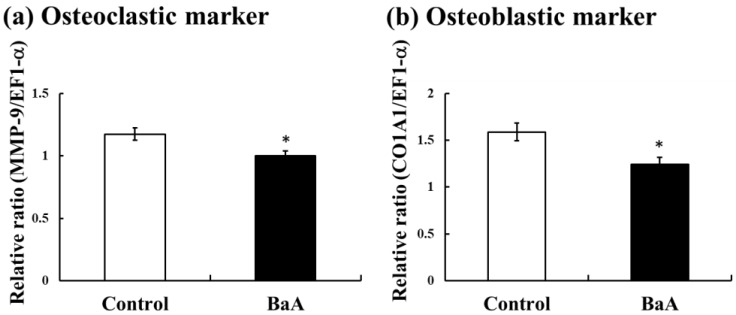
Effects of BaA on osteoclastic (**a**) and osteoblastic (**b**) marker messengerRNA (mRNA) expression in the scales of BaA-treated or untreated nibbler fish. BaA (10 ng/g body weight) was injected intraperitoneally (four times) into nibbler fish for 10 days during breeding. Thereafter, osteoclastic and osteoblastic marker mRNA expressions were examined in the control and experimental groups. * indicates a statistically significant difference at *p* < 0.05 from the values in the control scales (n = 8).

Osteoclastic marker (MMP9) mRNA expression results are shown in Figure 2a. BaA suppressed MMP9 mRNA expression. The mRNA expression of osteoblastic marker COL1A1 also decreased significantly (Figure 2b).

### 3.3. Effects of BaA on Total Protein and Albumin in The Plasma of BaA-Treated or Untreated Nibbler Fish

**Figure 3:**
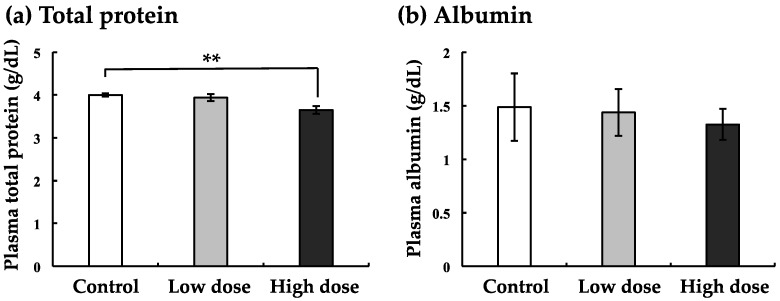
Effect of BaA on total protein (**a**) and albumin (**b**) in the plasma of BaA-treated or untreated nibbler fish. BaA (1 or 10 ng/g body weight) was injected intraperitoneally (four times) into nibbler fish for 10 days during breeding. Thereafter, total protein and albumin were examined in the control and experimental groups. ** indicates a statistically significant difference at *p* < 0.01 from the values in the control scales (n = 8). The detection limits of total protein and albumin were 1.0 and 1.0 g/dL, respectively.

In order to perform a comprehensive analysis of blood components, we first analyzed total protein and albumin in the plasma of nibbler fish. At low dose exposure, total protein levels in BaA-treated nibbler fish were not changed as compared to those in control nibbler fish (Figure 3a). However, a significant difference in the total protein levels between nibbler fish treated with the high dose of BaA and control nibbler fish was obtained (Figure 3a). Also, albumin levels tended to decrease when treated with low and high doses of BaA, although there was no significant difference in the albumin levels of BaA-treated and control groups (Figure 3b).

### 3.4. Changes in The Enzyme Markers of The Liver with BaA Treatment

**Figure 4:**
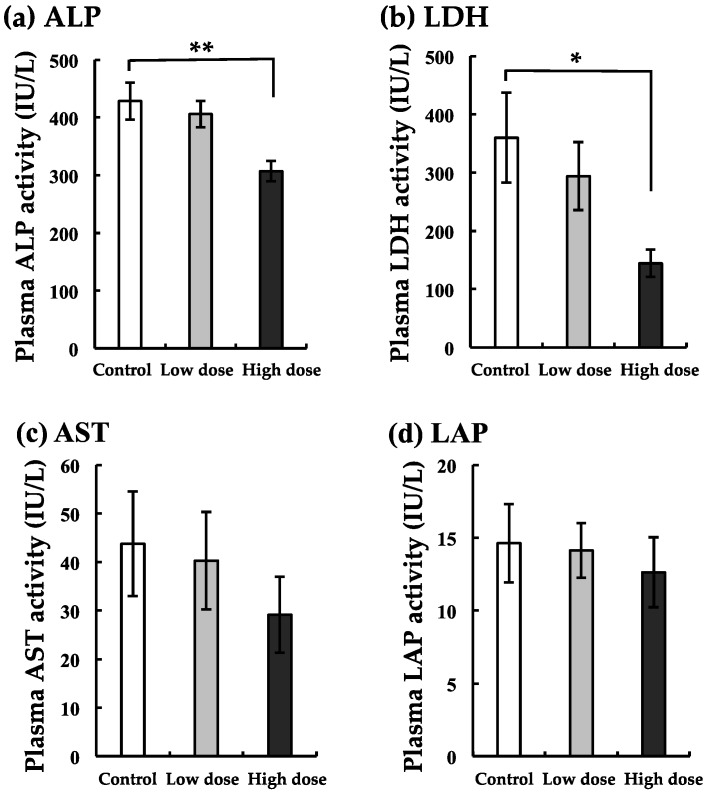
Changes in alkaline phosphatase (ALP) (**a**), lactate dehydrogenase (LDH) (**b**), aspartate transaminase (AST) (**c**), and leucine aminopeptidase (LAP) (**d**) of the liver with BaA treatment. BaA (1 or 10 ng/g body weight) was injected intraperitoneally (four times) into nibbler fish for 10 days during breeding. Thereafter, plasma liver markers were examined in the control and experimental groups. * and ** indicate statistically significant differences at *p* < 0.05 and *p* < 0.01, respectively, from the values in the control scales (n = 8). The detection limits of ALP, LDH, AST, and LAP were 2, 6, 3, and 0.4 IU/L, respectively.

We noted that the liver is a target organ for BaA because total protein and albumin decreased as shown in Figure 1. To examine the influence of BaA on liver diseases, the blood marker enzyme activities for ALP, LDH, AST, and LAP were measured in the plasma of BaA-treated nibbler fish or untreated nibbler fish. Data are shown in Figure 4.

In the low-dose group, all markers tended to decrease. Significant differences in the ALP and LDH of the plasma of BaA-treated nibbler fish as compared with that of untreated nibbler fish were observed at the high dose. The remarkable suppression of ALP was obtained at the high dose.

### 3.5. Changes in Markers of Lipid Metabolism with BaA Treatment

**Figure 5:**
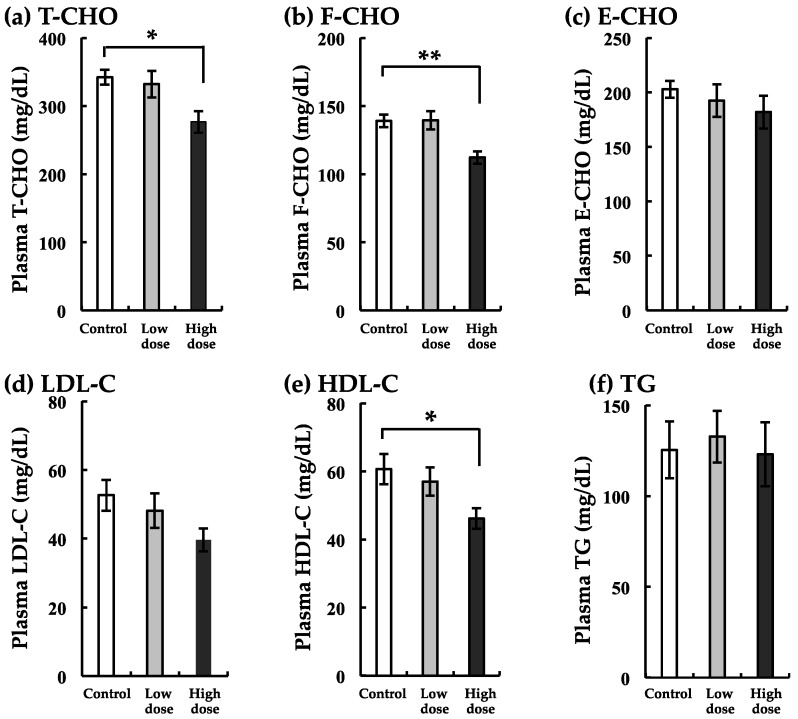
Changes in total-cholesterol (T-CHO) (**a**), free-cholesterol (F-CHO) (**b**), ester type-cholesterol (E-CHO) (**c**), low-density lipoprotein cholesterol (LDL-C) (**d**), high-density lipoprotein cholesterol (HDL-C) (**e**), and triglyceride (TG) (**f**) with BaA treatment. BaA (1 or 10 ng/g body weight) was injected intraperitoneally (four times) into nibbler fish for 10 days during breeding. Thereafter, plasma lipid markers were examined in the control and experimental groups. * and ** indicate statistically significant differences at *p* < 0.05 and *p* < 0.01, respectively, from the values in the control scales (n = 8). The detection limits of T-CHO, F-CHO, LDL-C, HDL-C, and TG were 1, 1, 1, 2, and 2 mg/dL, respectively.

In the low-dose group, T-CHO, E-CHO, LDH-C, and HDH-C tended to decrease, although there were no significant differences between the experimental and control groups (Figure 5). In the high-dose group, F-CHO levels were largely suppressed. Other markers (T-CHO and HDL-C) decreased significantly in nibbler fish treated with the high dose of BaA (Figure 5).

### 3.6. Changes in Na, K, and Cl Levels with BaA Treatment

**Figure 6:**
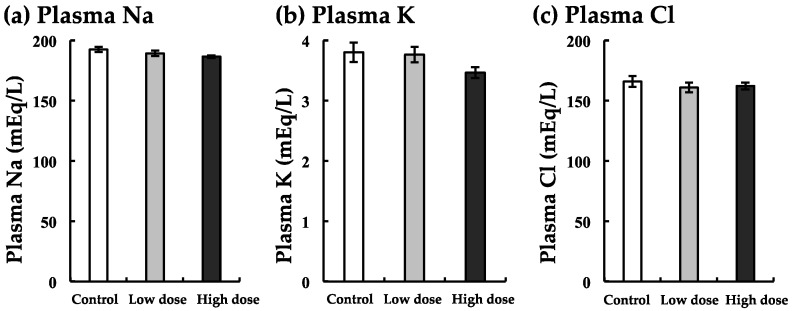
Changes in Na (**a**), K (**b**), and Cl (**c**) levels with BaA treatment. BaA (1 or 10 ng/g body weight) was injected intraperitoneally (four times) into nibbler fish for 10 days during breeding. Thereafter, plasma Na, K, and Cl were examined in the control and experimental groups. There was no significant difference between the control and experimental groups (n = 8). The detection limits of Na, K, and Cl were 10, 1, and 10 mEq/L, respectively.

Plasma Na, K, and Cl levels were decreased slightly with both low and high doses of BaA (Figure 6). In these minerals, there was no significant difference between the experimental and control groups (Figure 6).

## 4. Discussion

We are the first to demonstrate that BaA decreased both plasma Ca and Pi levels in marine teleosts. We previously reported that BaA induced hypocalcemia at 24 and 48 h after injection into goldfish (freshwater fish) [20]. In our previous study, we could not measure the plasma Pi level. It is well known that Ca and Pi are bone components. In addition to the plasma Ca concentrations, we found that the plasma Pi concentrations also decreased after injection of BaA into nibbler fish. Furthermore, the mineral concentrations of Na, K, and Cl did not change significantly with BaA treatment. Taking these results into consideration, BaA has a specific effect on bone mineral metabolism. Through these specific effects, we concluded that PAHs induced bone deformities in teleosts such as Pacific herring, pink salmon, and sea bass [5,25].

We discovered the toxicity of mono-hydroxylated polycyclic aromatic hydrocarbons (OHPAHs), metabolites of PAHs, in osteoclasts and osteoblasts of fish scales [17]. Namely, 4-hydroxybenz[*a*]anthracene (4-OHBaA) suppressed both tartrate-resistant acid phosphatase and ALP activities in the cultured scales of goldfish (freshwater teleost) and wrasse (marine teleost) [17]. In fact, 4-OHBaA, which is one of the metabolites of BaA by a conversion enzyme (P4501A1), was detected in the bile of goldfish at 12, 24, 48, and 72 h after the administration of BaA into these goldfish [20]. In nibbler fish, OHBaA, metabolites of BaA, suppressed osteoclasts and osteoblasts. The suppression of both an osteoclastic marker (MMP9) and an osteoblastic marker (COL1A1) coincided with the action of 4-OHBaA [17]. In mammals, PAHs (BaP and DMBA) present in cigarette smoke induced bone loss in an ovariectomized rat [2]. In addition, BaP inhibited osteoclastogenesis in rabbit osteoclasts and RAW264.7 cells (a mouse monocyte macrophage cell line) [26]. Although PAHs are known to bind to the aryl hydrocarbon receptor [6,26], their subsequent action mechanism is unknown. In mammals as well as teleosts, OHPAHs converted from PAH may disrupt bone metabolism. To elucidate the further mechanism of PAH toxicity on bone metabolism, we are planning to examine the influence of OHPAH on a mammalian osteoblastic cell line.

In order to perform a comprehensive analysis of blood components, we analyzed total protein and albumin in the plasma of nibbler fish. A significant difference in total protein levels between nibbler fish treated with the higher dose of BaA and control nibbler fish was obtained. Also, albumin levels decreased in a dose-dependent manner. Nutritionally, BaA may have adverse effects on fish because BaA acts on the liver metabolism, such as LDH and cholesterol metabolism. BaP has been identified as being highly carcinogenic [27,28]. BaP showed strong repression of genes involved in cholesterol and fatty acid biosynthesis [29]. However, BaA did not influence TG levels, although BaA decreased T-CHO, F-CHO, E-CHO, LDH-C, and HDH-C. The action mechanisms of BaP and BaA may thus be different. In addition, we found that ALP activity appeared to have decreased remarkably. ALP activity is also known as an osteoblastic marker [11,21,30], although ALP is a marker of liver diseases [31,32]. In the present study, we indicated that the mRNA expression of the osteoblastic marker COL1A1 decreased significantly. Under the influence of both bone and liver, ALP activity appeared to have decreased remarkably.

We can analyze the influence of BaA on osteoblasts and osteoclasts using in vitro cultured fish scales. The teleost fish has a unique hard tissue, scales that consist of osteoblasts, osteoclasts, and calcified bone matrix, including type 1 collagen, bone γ-carboxyglutamic acid protein, osteonectin, and hydroxyapatite [11,33]. In addition, teleost scales, like the mammalian endoskeleton, are known to work as a potential internal calcium reservoir [11,33]. From morphological observation, we demonstrated that the osteogenesis of regenerating scale is quite similar to that of mammalian membrane bone [34]. Furthermore, the fine structure of osteoclasts and osteoblasts in scales is similar to those found in mammals [24]. Using this in vitro system [35], the effects of endocrine disrupters, such as bisphenol-A [36], tributyltin [37], and polychlorinated biphenyl [38], and heavy metals (i.e., cadmium and mercury) [22,39,40], on osteoblasts and osteoclasts were measured. The concentration of cadmium (even at 10^−13^ M) influenced osteoclastic activity in the scale [39]. Recently, we demonstrated the toxicity of gadolinium (Gd) on osteoclasts and osteoblasts of goldfish scales [41]. Even Gd at a concentration of 10^−13^ M suppressed osteoclastic activity at 6 h of incubation. Osteoblastic activity was also suppressed by Gd in the concentration range of 10^−10^ to 10^−6^ M at 6 h of incubation [41]. Therefore, the toxicity of Gd to osteoclasts was comparable to that of Cd. The osteoblastic inhibition of Gd was also almost equal to that of tributyltin (10^−10^ to 10^−5^ M) [37]. Considering the results above, fish scales are very useful for evaluating the effect of environmental pollutants on bone metabolism.

In the present study, we found that a low concentration of BaA has toxicity for the bone and liver of nibbler fish (marine teleost). As highly polluted seawater in the natural environment included BaA [18], we call for the prevention of PAH pollution.

## 5. Conclusions

In an in vivo experiment with nibbler fish (marine teleost) injected intraperitoneally with a low concentration of BaA (1 or 10 ng/g body weight), we found that BaA suppressed plasma Ca and Pi levels resulting from the inhibition of osteoclasts and osteoblasts. Furthermore, we indicated that total protein, metabolic enzymes in the liver, and T-CHO, F-CHO, and HDL-C levels significantly decreased in the BaA-injected fish. These results indicate that BaA may affect liver diseases in addition to bone diseases. As BaA is included in highly polluted seawater [18], we should emphasize the prevention of aquatic PAH pollution.

## Figures and Tables

**Table 1:** Primer sequences for real-time quantitative PCR.

Name	Forward Primer	Reverse Primer	Accession Number
MMP-9	TGTGGTGCTCAACCACCTACAACT	ATCCCTGCCTTGAGTGGTGCAT	LC198841
COL1A1	GTGAGGTCGCCAAGAAGAAC	ATGAGACGCAGGAAGGTCAG	AB874603
EF-1α	GTATGGTCGTCACCTTTGCTC	GTGGGTCGTTCTTGCTGTC	AB874605

MMP-9: matrix metalloproteinase-9; COL1A1: collagen, type 1, α1; EF-1α: elongation factor-1α.
